# Large-Truck Safety Warning System Based on Lightweight SSD Model

**DOI:** 10.1155/2019/2180294

**Published:** 2019-10-13

**Authors:** Dong Xiao, Hongzong Li, Chenyi Liu, Qifei He

**Affiliations:** ^1^Information Science and Engineering School, Northeastern University, Shenyang 110819, China; ^2^Liaoning Key Laboratory of Intelligent Diagnosis and Safety for Metallurgical Industry, Northeastern University, Shenyang 110819, China; ^3^Intelligent Mine Research Center, Northeastern University, Shenyang 110819, China; ^4^Computer Science and Engineering School, Northeastern University, Shenyang 110819, China

## Abstract

Transportation is an important link in the mining process, and large trucks are one of the important tools for mine transportation. Due to their large size and small driving position, large trucks have a blind spot, which is a hidden danger to the safe transportation of mines and has a great impact on production efficiency and economic loss. The traditional large truck safety warning system mainly uses the ultrasonic short-distance ranging method, radar ranging method, GPS (Global Positioning System) technology, and so on. The disadvantage of these methods is that they are affected by the environment and weather, and they cannot display the object status in real time. Therefore, it is becoming increasingly important to realize the large truck safety warning system based on machine vision. Therefore, this paper proposes a lightweight SSD (Single Shot MultiBox Detector) model and an atrous convolution to build a large-truck object recognition model. First, the training images are collected and marked. Then, the object recognition model is established by using the lightweight SSD model. The atrous convolutional layer is introduced to improve small object detection accuracy. In the end, the objectness prior method is used to improve the classification speed. Experimental results show that, compared with the original SSD model, the lightweight SSD model occupies less space and runs faster. The lightweight SSD model with the atrous convolutional layer is more sensitive to small objects and improves detection accuracy. The objectness prior method further improves the identification speed. Compared with the traditional large truck safety warning, the system is not affected by the environment and realizes the visualization of large truck safety warning.

## 1. Introduction

With the continuous improvement of mining technology and the continuous increase of mining intensity [1], the large scale of equipment is also developing, and the large truck is one of the main tools for mine production and transportation. There are many characteristics of large mining trucks. The mine transportation road is complex, with more curves and slopes, and the roads are often changed, and the working environment is bad. There are many kinds of auxiliary vehicles, such as command cars, gunpowder cars, bulldozers, sprinklers, etc. Large trucks are bulky, and the cab is only a small part of the upper left. There are blind spots in most parts of the right side of the body and the front and back parts of the vehicle. The driver is working hard, driving for hours on end, and is often bored. The mining area is working day and night, and the vehicle sizes are mixed [2]. The above characteristics lead to the occurrence of large-truck collision accidents and become a major hidden danger to the safety transportation of mines, which has a great impact on production efficiency and economic loss. In China, there have been related studies on the safety warning of mine trucks [3]. Ultrasonic short-distance ranging methods [4], radar ranging methods [5], and GPS (Global Positioning System) technology [6] have been widely used [7, 8]. The transmission speed of the ultrasonic short-distance ranging method is very easily affected by the weather and cannot achieve the ideal effect in bad weather. The radar ranging method is weak in electromagnetic interference. GPS technology is mature and widely used, but some deep open-pit mines using GPS technology for a safety warning device in the satellite system signals sometimes have a blind area causing positioning failure and early warning failure. These traditional methods cannot show the object status in real time, which can easily lead to misjudgment and miscalculation. Therefore, the development of the visualization of large truck hazard warning systems is an important problem to be solved in mine safety transportation.

In recent years, the deep neural network (DNN) has achieved great success in the field of object recognition [9]. Unlike traditional manual extraction features, deep neural networks can learn useful features by constructing models with multiple hidden layers and massive training data. It is adaptable and can achieve better performance in practice. Girshick et al. put forward a kind of candidate region and convolution neural network (CNN), in combination with the regional convolution neural network model (R-CNN) [10] for the object detection and classification, that became a staple of using deep learning for object detection. On this basis, Fast R-CNN [11] and Faster R-CNN [12] have been successively launched, and the R-CNN series model has achieved great success and been further improved. Although the R-CNN model has high accuracy, it is too expensive and too slow to be used for real-time object detection. To overcome this problem, Redmon et al. put forward a method using a novel YOLO (You Only Look Once) [13] detection model forecast object box directly. It is simpler, faster, and more accurate than the R-CNN series model. Liu et al. further proposed an SSD (Single Shot MultiBox Detector) [14] model to eliminate the regional proposal and subsequent resampling stages to obtain higher accuracy and faster speed compared to the YOLO model.

The SSD model has many features. First, the convolution layer is used to replace the whole connection layer of Faster R-CNN to form the network structure of full convolution so that the object detection speed is greatly improved. Second, the proposed mechanism of Faster R-CNN is changed into the anchor box generation mechanism, which improves object detection accuracy. However, SSD models have limited ability to detect small objects. The SSD model adopts multiscale feature fusion technology to realize the detection of different size objects. The low-level feature graph contains the characteristics of small objects, but the contextual semantics [15] are not enough. The high-level feature graph is rich in contextual semantics, but the characteristics of the small objects are very small. The atrous convolution [16] makes the feature graph no longer shrink after convolution and pooling while ensuring that the feeling field remains unchanged. It makes the small objects feature coexist with the contextual semantic information, which is beneficial for small object detection.

Transfer learning [17], as the name suggests, involves migrating trained model parameters to new models to help train new models. Because most of the data correlates with the task, the model parameters are learned through the transfer of learning to share with the new model and speed up and optimize the learning efficiency of the model rather than learning from zero similar to most network learning. The SSD model is a type of deep learning model, and deep learning models have a strong demand for training data [18]. In the case of a small dataset, model performance can drop dramatically. To solve this problem, the learning weight is migrated to the lightweight SSD model from the pretrained model (for example, VGG-16 [19]) and fine-tuning the dataset from the truck perspective training dataset.

In this paper, a lightweight SSD model based on atrous convolution is proposed to realize object recognition from the perspective of the truck. The identification of small objects is enhanced while minimizing the volume of the model. On this basis, the objectness prior method is added to realize the visualization of the large truck hazard warning system. In the training process of the model, it is helpful to improve the training speed and precision of the model by adopting the “objectiveness” of the original model.

## 2. Data Collection and Labeling

The dataset in this paper is from the PASCAL VOC 2012 dataset, KITTI dataset, and more than 10,000 images taken from the truck's perspective. PASCAL VOC is a standard dataset for visual object classification and detection [20]. It provides a collection of 20 categories (pedestrians, vehicles, animals, etc.). The KITTI dataset [21] is the largest computer vision algorithm evaluation dataset in the world. It contains real image data collected from scenes such as urban, rural and motorway, with up to 15 cars and 30 pedestrians in each image, as well as varying degrees of occlusion. The collected images are RGB images (RGB is the color of red, green, and blue channels), the resolution is 1920 × 1080 pixels, and the set contains the objects to be identified: pedestrians and various vehicles.

**Figure 1:**
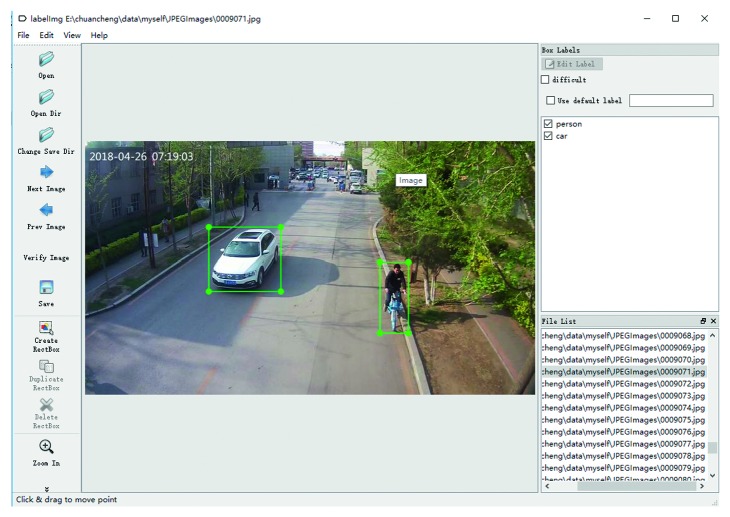
LabelImg software operation interface.

First, PASCAL VOC data is set aside from categories such as person and car. The TFRecord file required to train the TensorFlow framework [22] is generated together with the image file and the XML file. The object information document file of the KITTI data set is then converted into an XML file corresponding to the image. The TFRecord file is generated together with the image file and the XML file. Finally, the collected images are processed. Zooming in on the image: Zooming the picture to 711 × 400 pixels makes it easy to train the model. Mark the pictures: LabelImg software is an image annotation tool used to annotate target object category information and location information in the original image. The corresponding XML file is generated for each image to record the object classification information, object location information, and the remaining basic information of the image. Two diagonal coordinates (the upper left corner and the lower right corner) are located for the target. There are two types of labeling categories: person and car. For images with multiple objects, each target is marked individually. After the annotation is completed, an XML file with the same name will be generated. The TFRecord file is generated together with the image file and the XML file.  Figure 1 shows the LabelImg software operation interface.

## 3. Establishment of the Object Recognition Model

The model of this paper is based on the convolution neural network. A lightweight SSD model, based on atrous convolution, is proposed. This model removes redundant feature layers and forms a lightweight model based on the original SSD model. At the same time, the atrous convolutional layer is used to add the lower feature layer to multiscale feature fusion, which is helpful for the detection of small objects. Finally, this model introduces the objectness prior algorithm to improve the model detection speed.

### 3.1. Convolutional Neural Network

The convolutional neural network is a multilayer perceptron. It includes the input layer, the hidden layer, and the output layer, and the hidden layer is divided into the convolution layer and the pooling layer [23]. The image is input in matrix form, and the desired data is output by the convolution layer and pooling layer. The convolution layer implementation process is as follows: a trainable filter is used to convolve an input image, plus an offset, and the convolution layer is finally obtained by the activation function. Assuming that the *l* layer is the convolution layer, the specific calculation of the *j*th feature mapping is as follows:

(1) $$\begin{array}{c} x_{j}^{l}=f\left(\underset{i\in M_{j}}{\sum }x_{i}^{l-1}\;*\;k_{ij}^{l}+b_{j}^{l}\right), \end{array}$$

where *f* is a nonlinear activation function (e.g., sigmoid function, ReLU function, tanh function, etc.) *M*_*j*_ is the input data matrix, *k*_*ij*_^*l*^ is the convolution kernel of the *i*th data of layer (l−1) and the *j*th data of the *l* layer, and *b*_*j*_^*l*^ is the offset.

The realization process of the pooling layer is to transform the *m* pixels of the neighborhood into a pixel by the sampling function, then by the scalar weighting, and then, by an offset, the pooling layer is finally obtained by the activation function. Assuming that the *l* layer is the pooling layer, the specific calculation of the *j*th feature map is as follows:

(2) $$\begin{array}{c} x_{j}^{l}=f\left(\beta_{j}^{l}\text{down}\left(x_{i}^{l-1}\right)+b_{j}^{l}\right), \end{array}$$

where *f* is a nonlinear activation function, *β*_*j*_^*l*^ is a weighted scalar, down is the sampling function (such as random sampling, mean sampling, maximum sampling, etc.), and *b*_*j*_^*l*^ is the offset.

### 3.2. Atrous Convolution

The atrous algorithm refers to the convolution operation after inserting the appropriate number of zeros between the filter points. This technique has a long history in signal processing, which was originally used to improve the computational efficiency of nonextracted wavelet transform [24] and was also used in the deep convolutional neural network. This algorithm can calculate the response at any level of resolution.

**Figure 2:**
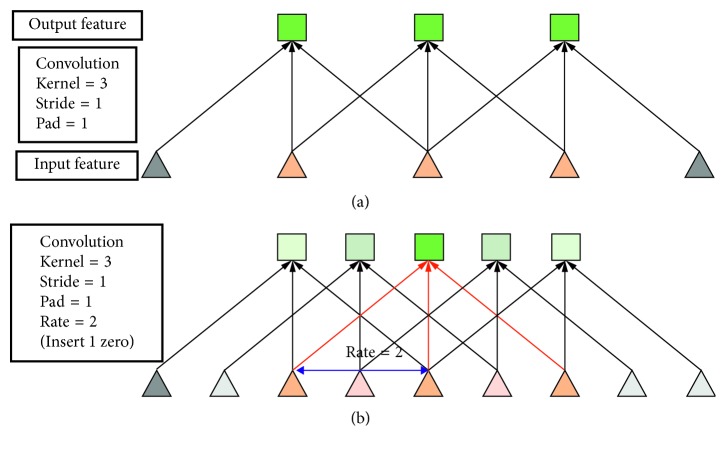
One-dimensional signal feature extraction: (a) standard convolution and (b) atrous convolution.

First, consider the case of atrous convolution with a one-dimensional signal. Assuming that the one-dimensional input signal is *x*[*i*] and the output of the atrous convolution is *y*[*i*], the calculation formula is as follows:

(3) $$\begin{array}{c} y\left[i\right]=\overset{K}{\underset{k=1}{\sum }}x\left[i+r\cdot k\right]w\left[k\right], \end{array}$$

where *w*[*k*] is a filter, *K* is the filter length, and *r* is the proportional parameter representing the input signal sampling step.  Figure 2 shows the concrete realization of the convolution of the one-dimensional signal.  Figure 2(a) shows the sparse feature extraction process of standard convolution in low resolution input.  Figure 2(b) shows the dense feature extraction process of atrous convolution in high resolution input.

**Figure 3:**
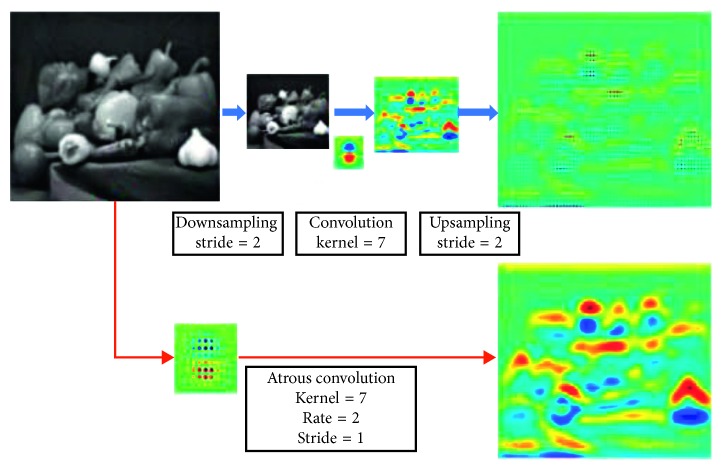
Two-dimensional signal feature extraction.

The realization process of convolution processing in a two-dimensional image is shown in Figure 3. The top line of Figure 3 is the standard convolution sparse feature extraction. The bottom line of Figure 3 is the dense feature extraction of the atrous convolution with ratio *r* equal to 2. Given a picture, standard convolution is applied. The first step is to reduce the resolution with the lower sampling operation of 2, and then the convolution operation is performed with a Gaussian kernel of 7 × 7, and the characteristic graph obtained at this time is only one-fourth the size of the original image. The image is treated with an atrous convolution. The rate of the convolution kernel is sampled at 2, and 0 is filled in the vacancy. A convolution operation is done using the new convolution kernels, and the resulting feature map is the same size as the original image. Although the size of the convolution kernel is increased, only the nonzero values of the convolution kernel need to be considered, so the convolution kernel parameters and the operation number of each position remain the same.

The atrous convolution, with a rate of *r*, joins (r−1) zero value between any two adjacent values of the convolution kernel. It effectively expands the convolution kernel without increasing the number of parameters and the amount of computation. Therefore, it provides an effective mechanism to control the field of vision, achieving the best balance between small object feature extraction and contextual semantics. For a convolution kernel of *k* × *k*, the expanded size is

(4) $$\begin{array}{c} k_{e}=k+\left(k-1\right)\left(r-1\right). \end{array}$$

### 3.3. Lightweight SSD Model

Because the object in the truck view is smaller, this paper removes the extra middle layer used to detect large objects in the SSD model and constructs a lightweight model [25], reducing the model volume and improving the detection speed. The SSD model is a method to realize object detection and recognition using a single deep neural network model. This method is a comprehensive approach to the anchor box of Faster R-CNN and a single neural network of YOLO. It has both Faster R-CNN accuracy and YOLO detection speed, which can achieve high-accuracy real-time detection.

**Figure 4:**
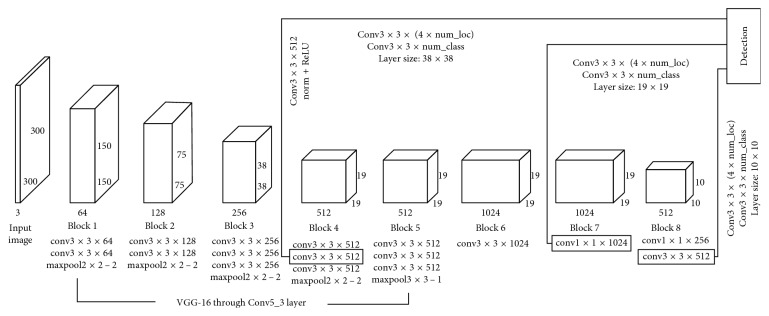
Lightweight SSD model extraction.

The lightweight SSD model structure is shown in Figure 4. Each input image is cut out for an average of a 300 × 300 × 3 tensor. Then, a series of feedforward convolutional layers are applied to the input image. The bottom of the network (block 1 to block 5) is based on the VGG-16 model. In contrast to the original SSD model, the lightweight model eliminates a number of middle layers, and therefore, it is smaller.

To perform object detection on multiple scales, the feature graph was extracted from block 4 (38 × 38), block 7 (19 × 19) and block 8 (10 × 10). For each feature graph on each block, it contains a specific number of anchor boxes. These anchor boxes have a certain ratio and aspect ratio. Two different filter types are applied to each feature graph to predict bounding box offset and object score.

Between block and block, the feature extraction and information transfer are carried out by the convolution layer and pooling layer. Take block 1 as an example, the input image size of 300 × 300 × 3 is processed twice by 64 convolution kernels of 3-by-3, and 64 characteristic layers of 300 × 300 pixel are obtained. After the maximum pool layer, the characteristic layer becomes the original half, and 64 characteristic layers with a size of 150 × 150 are obtained.

### 3.4. A Lightweight SSD Model Based on Atrous Convolution

There are many small objects in the truck view, and the SSD model has limited ability to detect small objects. Atrous convolution enables the feature graph to combine small object characteristics and contextual semantic information, which is beneficial to the detection of small objects. In this paper, a lightweight SSD model, based on atrous convolution, is proposed to balance small object feature information and contextual semantic information to achieve small object detection.

**Figure 5:**
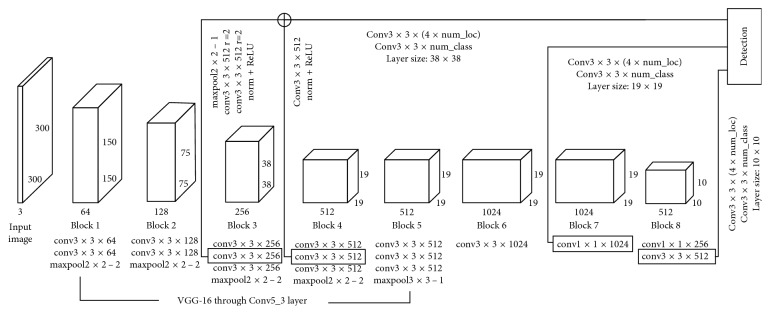
A lightweight SSD model based on atrous convolution.

The lightweight SSD model based on the atrous convolution is shown in Figure 5. The model extracted features of conv3_3 and conv4_3 in the VGG-16 basic network. Conv3_3 layer passes a maximum pooling layer with a step length of 1. It then goes through the two convolution layers where the convolutional kernel is 3 × 3 and the rate is 2 (the atrous convolution rate is 2). Finally, the normalized layer is passed. The conv4_3 layer, after the normalization layer is connected with the treated conv3_3 layer, is the new block. The new block is extracted with a multiscale feature with block 7 and block 8.

### 3.5. Objectness Prior

Each feature map has a specific number of anchor boxes as candidate areas, but only a small portion of the anchor box actually contains the objects. Therefore, the number of objects and nonobjects is extremely unbalanced resulting in a serious imbalance of positive and negative sample proportions. This paper uses objectness prior [26] to filter most of the negative samples.

**Figure 6:**
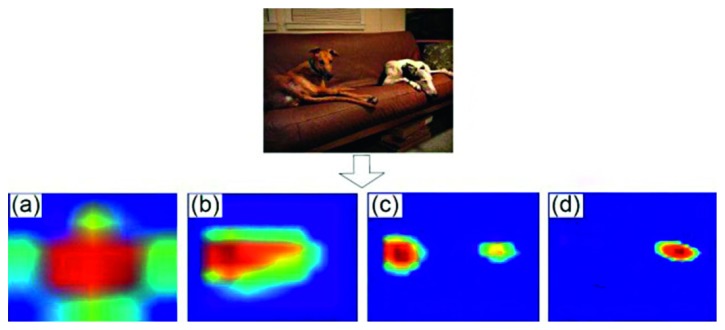
Multiscale objectness prior.

First, a binary tag is assigned to each candidate area to determine if the candidate area contains objects. If the candidate area contains an object, a category label is specified to determine the object category. Otherwise, it is the background. The object search space is greatly reduced.  Figure 6 shows a multiscale objectness prior graph generated from a specific image. For visualization, the average of multiple objectness prior feature graphs is taken along the channel direction. Figures 6(a) and 6(b) highlight the position of the sofa.  Figure 6(c) highlights the brown dog's position.  Figure 6(d) shows the position of the white dog. It can be seen that the objectness prior graph can clearly indicate the existence of objects to be detected. Therefore, the search scope can be greatly reduced, and different scale objects will be reflected in the corresponding feature graph.

## 4. Experiment

### 4.1. Evaluation Standard

To judge a model, it is necessary to evaluate its positioning and classification accuracy. Because of the different emphasis, the object detection has many evaluation indexes. We use the most commonly used measurement indexes in the field of detection, mean average precision (mAP), to evaluate model performance in this paper. The average precision (AP) is calculated to evaluate model performance in each category. After obtaining the AP values of each category, the mAP, which is the average of all categories, is used to evaluate the model performance in all categories. The calculation process is as follows: first, calculate the average precision of each category:

(5) $$\begin{array}{c} P=\frac{1}{R}\overset{n}{\underset{j=1}{\sum }}I_{j}\frac{R_{j}}{j}, \end{array}$$

where *R* represents the number of all related objects in a category of datasets and *n* represents the number of objects in the dataset. If the *j*th object is related, *I*_*j*_ is 1. Otherwise, *I*_*j*_ is 0. *R*_*j*_ is the number of related objects in the former *j* objects. The mean of the average precision of multiple categories is the mAP. The mAP value is between 0 and 1, and the larger the value, the higher the model detection precision.

This paper uses FPS, frames per second, to evaluate the speed of the model.

### 4.2. Experiment Part

The experimental hardware environment is the computer, which has an Intel Core i5-7500 processor, an NVIDIA GTX 1050 Ti graphics card, and 64 GB RAM. The software environment is a Windows 10 64-bit system with Python, cuda, OpenCV, and TensorFlow framework. Cuda accelerates image processing and OpenCV is used for real-time image display. The code implementation uses the TensorFlow framework and references the open source code of the SSD paper.

The training of the lightweight SSD model was mainly carried out in the PASCAL VOC 2012 dataset and KITTI data set, as well as 80% of the self-collected datasets. The remaining 20% of the collected datasets are used to validate and evaluate the model. The specific training steps are as follows: load the pretraining weights of the bottom of the model based on the VGG-16 model. Train other layers of lightweight models in the PASCAL VOC 2012 dataset and KITTI datasets. Then, the model is fine-tuned on the self-collected dataset.

In training, the Jaccard similarity between the ground truth box and the default candidate area generated by the model is used as the standard to distinguish the positive and negative samples. If the Jaccard similarity is greater than the set threshold of 0.5, it is set as a positive sample. Otherwise, it is a negative sample. When predicting, the default candidate area with the Jaccard similarity greater than the threshold of 0.5 is used as the object boxes. In addition, nonmaximum suppression (NMS) is used to eliminate similar borders.

This paper uses the root mean square prop (RMSProp) training model. RMSProp momentum is 0.9. RMSProp decay is 0.9. The parameters are randomly initialized. The initial learning rate is set at 0.0001. The learning rate decay factor is 0.94. Weight decay is 0.0004. The batch size is 24. In training, increasing the number of training times can help improve the accuracy of the model. In this paper, we trained 40,000 times in the PASCAL VOC 2012 dataset and KITTI dataset and then trained 40,000 times on the self-collected dataset.

## 5. Results and Analysis

In this paper, three sets of comparison experiments were carried out based on the selection of the small object feature extraction layer, the expansion rate of the atrous convolution, and the different models. The test results of the lightweight SSD model based on atrous convolution were tested and verified.  Section 5.1 compares the object feature extraction ability of different model characteristics. In Section 5.2, the effect of different expansion rates on object feature extraction is compared according to the difference of the expansion rate in the atrous convolution.  Section 5.3 is a comparison of the detection accuracy and speed of several SSD correlation models.

### 5.1. Comparison of Different Feature Extraction Layers

The resolution of the model lower layer is high. The small object feature is rich in information. Multiscale feature fusion with different feature layers will have different effects. The standard SSD model uses the conv4.3 layer to extract small object features. In this paper, after the atrous convolution and the conv4.3 layer are merged together, the conv3.3 layer is used as the feature extraction layer of the small object. In this section, we do an experiment by using different features to extract small object features.  Table 1 shows the comparison of the results of the SSD model using different feature extraction layers for the detection of truck view images.

In theory, the lower layer of the model can provide abundant feature information for object detection, but the feature extraction capability does not increase linearly. As seen in Table 1, the single-layer extraction feature conv4_3 layer is the strongest. The mAP of the conv4_3 layer is 59.3%. It is the design of the standard SSD model. Conv5_3 is the worst. Several feature layers are simply linked together to extract small object features. It can be seen that the combination of conv3_3 layer and conv4_3 layer can improve the accuracy of the model. In this paper, after the addition of atrous convolution, the feature layer is connected with other feature layers as the feature extraction layer of the small object, which can take into account the context semantic information and small target features. The detection accuracy can reach 64.7%, which is the best among several comparative experiments.

As seen from the aspects of detection speed, single-layer or multilayer feature layers that have direct links provide features that do not add additional computation, and the detection speed remains at 19 frames/s. In this paper, the model design, the atrous convolution, and normalization operation will increase the calculation amount of the model, which will reduce the detection speed to 17 frames/s.

### 5.2. Contrast of Atrous Convolution of Different Rates

Atrous convolution enables the feature graph to accommodate both small object feature information and contextual semantic information and is good for detecting small objects. The atrous convolution layer, with different expansion rates, can extract the characteristics of different size objects. In this section, the characteristic graph is processed by the atrous convolutional layer with different expansion rates, and the detection results are compared.  Table 2 shows the comparison of the results of the model with different expansion rates of the atrous convolution to the image of the truck.

Table 2 shows that the detection accuracy of the two-layer atrous convolution with an expansion rate of 2 is the highest. The detection accuracy is 64.7%. The detection accuracy of the two-layer atrous convolution with an expansion rate of 4 is the lowest. The detection accuracy is 62.5%. It can be seen that adding porous convolution can improve the detection accuracy, but the expansion rate of atrous convolution and the number of layers are not proportional to the detection accuracy of the model.

From the aspect of detection speed, changing the expansion rate and layer number of the atrous convolution has little effect on the detection speed. Considering the two aspects, this paper uses two-layer atrous convolution, with an expanded rate of 2, to extract the small object features from the perspective of the truck.

### 5.3. Comparison of the SSD Correlation Model

In this paper, the standard SSD model is improved, the redundant feature layer is removed, and the lightweight model is formed. The atrous convolutional layer is added to realize the detection of small objects. In this section, according to the improvement of the model, the SSD correlation model, the standard SSD model, the lightweight SSD model, and the lightweight SSD model based on the atrous convolution are compared.  Table 3 compares the detection effects of the SSD correlation model on truck view images.

As seen from Table 3, among the three models, the lightweight SSD model, based on atrous convolution in this paper, has a maximum detection accuracy of 64.7%. The detection speed is 17 frames/s. The detection speed of the lightweight SSD model can be as high as 19 frames/s. Compared with the standard SSD model, the detection speed of the lightweight SSD model is improved, but the detection accuracy is decreased. The lightweight SSD model, based on atrous convolution, will reduce the detection speed and improve the detection accuracy based on the lightweight model. Compared with the standard SSD model, the lightweight SSD model, based on atrous convolution in this paper, has improved the detection accuracy on the basis of keeping the detection speed constant.

### 5.4. Detection Effect of Lightweight SSD Model Based on Atrous Convolution

**Figure 7:**
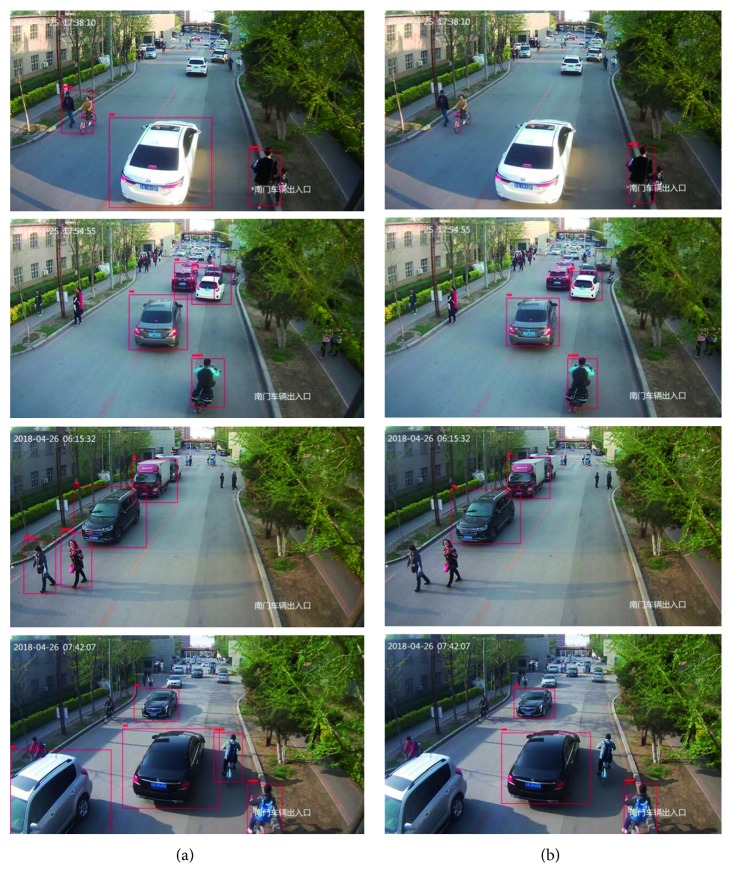
Test results of the standard SSD model and lightweight SSD model based on atrous convolution: (a) standard SSD model and (b) lightweight SSD model.

Figure 7 shows a comparison of the experimental results in the detection of truck view images of the standard SSD model and the lightweight SSD model, based on the atrous convolution in this paper. The left of figure shows the detection results of the original model, and the right of figure shows the detection results of the model in this paper. The results of the first group and the third group show that the new model can detect two more people than the original model. The second group shows that the new model can detect one more car than the original model, and the fourth group shows that the new model can detect one more person and one more car than the original model. The test results show that the new model can detect smaller targets than the original model and improve the detection accuracy of the occluded objects. The standard SSD model sets the object in the picture as the background. The new model of this paper can accurately locate and classify it.

## 6. Conclusion

This paper presents a lightweight SSD model based on atrous convolution. It is used in large-truck safety warning systems. On the basis of the standard SSD model, the model has removed the superfluous feature layer used to extract the larger target, which reduces the model volume and improves the detection speed. On this basis, the model is added to the atrous convolutional layer so that the feature graph contains both the contextual semantic information and the small object feature information. It improves the accuracy of small object detection. To further improve the detection speed, the model introduced the objectness prior method and adjusted the problem of the imbalance of positive and negative samples. The experimental results show that the new model proposed in this paper is better than the standard SSD model in detecting the truck image. It makes the large truck safety warning system play an important role. However, due to the limitation of the hardware condition, the detection speed of this model is somewhat unsatisfactory, and the next step is to improve the detection speed of the model.

## Figures and Tables

**Table 1:** The model test results with different feature layers.

Feature extraction layer	Fps (frames/s)	mAP (%)
conv3_3	19	58.9
conv4_3	19	59.3
conv5_3	19	58.2
conv3_3 + conv4_3	19	59.6
conv4_3 + conv5_3	19	58.7
conv3_3 + conv4_3 + conv5_3	17	59.1
Atrous conv3_3 + conv4_3	17	64.7

**Table 2:** The model test results with different atrous convolution rates.

Rate of atrous convolution	Fps (frames/s)	mAP (%)
2 × conv (rate = 2)	17	64.7
3 × conv (rate = 2)	17	63.8
2 × conv (rate = 3)	17	64.1
2 × conv (rate = 4)	17	62.5
2 × conv (rate = 6)	17	63.1

**Table 3:** SSD correlation model test results.

Object recognition model	Fps (frames/s)	mAP (%)
SSD300	17	62.0
Lightweight SSD300	19	59.3
Lightweight SSD300 + atrous	17	64.7

## Data Availability

The data used to support the findings of this study are available from the corresponding author upon request.
